# The Analysis of Two BDNF Polymorphisms G196A/C270T in Chinese Sporadic Amyotrophic Lateral Sclerosis

**DOI:** 10.3389/fnagi.2017.00135

**Published:** 2017-05-10

**Authors:** Lianping Xu, Danyang Tian, Jiao Li, Lu Chen, Lu Tang, Dongsheng Fan

**Affiliations:** Department of Neurology, Peking University Third HospitalBeijing, China

**Keywords:** amyotrophic lateral sclerosis, brain-derived neurotrophic factor, Chinese cohort, polymorphisms, susceptibility

## Abstract

Amyotrophic lateral sclerosis (ALS) is an ethnically heterogeneous motor neuron disease that results from the selective death of motor neurons in the brain and spinal cord. Brain-derived neurotrophic factor (BDNF) is widely distributed across the central and peripheral nervous systems and plays neurotrophic and other physiological roles in various brain regions. Alterations of neurotrophin availability have been proposed as a pathogenic mechanism underlying ALS neurodegeneration. Several genetic studies have shown a significant association between schizophrenia, Alzheimer's disease, and Parkinson's disease and certain BDNF polymorphisms, specifically G196A (rs6265) and C270T (rs56164415). However, the relationship between the G196A and C270T polymorphisms and ALS has never been investigated. We hypothesized that sporadic ALS (sALS) and disease susceptibility could arise due to BDNF polymorphisms and investigated the relationship between ALS and the BDNF polymorphisms G196A and C270T in a large Chinese cohort. We demonstrate that the frequency of the CT genotype and of the C270T T allele was significantly higher in the ALS group than in controls, although G196A was not associated with sALS. These data provide the first demonstration that the BDNF C270T polymorphism may be a candidate susceptibility locus for sALS, at least in Han Chinese.

## Introduction

Amyotrophic lateral sclerosis (ALS) is an ethnically heterogeneous motor neuron disease that results from the selective death of motor neurons in the brain and spinal cord (Chen et al., 2015; Huynh and Kiernan, 2015; Shahrizaila et al., 2016). The crude annual incidence rate of ALS in the general European population was 2.16 per 100,000 person years (Logroscino et al., 2010). There are several phenotypes of ALS, including limb-onset ALS, bulbar-onset ALS, progressive muscular atrophy (PMA), primary lateral sclerosis (PLS), and several regional limb variants, such as flail-arm syndrome (FAS). The pathogenesis of ALS is unclear. Approximately 90% of ALS cases are sporadic (sALS), while approximately 10% of cases are familial (fALS). The heritability of ALS is high; twin studies have estimated the genetic component to be 0.61 and the unshared environmental component to be 0.39 (Al-Chalabi et al., 2010). Based on the hypothesis that sporadic disease may arise from complex interactions between genetic susceptibility and the environment, the scientific community began to explore sALS susceptible genes. Several previous studies have identified different susceptibility genes in different ethnic cohorts, including DPP6, ITPR2, UNC13A, FGGY, ELP3, KIFAP3, 9p21.2, ZNF512B, TIMA1, and SCNN1A (Van Es et al., 2007, 2008, 2009a; Cronin et al., 2008; Landers et al., 2009; Simpson et al., 2009; Van Es et al., 2009b; Iida et al., 2011; Chen et al., 2014). However, it is believed that there are more susceptible genes need to be identified to help better understand the pathophysiology of sALS.

Brain-derived neurotrophic factor (BDNF), as a member of the neurotrophin family of growth factors, is widely distributed across the central nervous system and plays neurotrophic and other physiological roles in various brain regions (Leibrock et al., 1989; He et al., 2013). Defective BDNF expression and/or function is thought to be associated with depression, schizophrenia, Alzheimer's disease (AD), Parkinson's disease (PD), and ALS (Hyman et al., 1991; Phillips et al., 1991; Holsinger et al., 2000; Castrén et al., 2007; Favalli et al., 2012; Libman-Sokołowska et al., 2015). Alterations of neurotrophic availability have been proposed as a pathogenic mechanism underlying ALS neurodegeneration (Duberley et al., 1997; Kawamoto et al., 1998; Nishio et al., 1998; Tremolizzo et al., 2016). Although therapeutic trials of BDNF infusion have failed to show significant clinical benefit (1999; Ochs et al., 2000). BDNF has been shown to impact the survival of motor neurons in both *in vitro* and *in vivo* models of neuron injury or death (Sendtner et al., 1992; Henderson et al., 1993; Koliatsos et al., 1993; Mitsumoto et al., 1994; Ikeda et al., 1995). Transplantation of a mixture of such MPC populations (BDNF, GDNF, VEGF, IGF-1) into the hind legs of SOD1 G93A transgenic mice (SOD1 mice), the commonly used model of ALS, delayed the onset of disease symptoms by 30 days and prolonged the average lifespan by 13 days (Dadon-Nachum, 2015). Treated mice also showed a decrease in the degeneration of neuromuscular junction and an increase in axonal survival (Deepa et al., 2011). These information indicated that BNDF may play an important role in pathophysiology of sALS. So we hypothesized that some polymorphisms of the BNDF gene resulted in deceased neurotrophic availability and/or changed physiological roles may increase susceptibility to sALS.

Functional polymorphisms of the BDNF gene have been investigated in AD, PD, schizophrenia, depression patients, such as rs2030324, rs2049045, rs6265, rs2203877, rs7103411, rs988748, rs6265 [G196A], rs56164415[C270T], rs16917204 [G11757C], rs13306221 [G-712A] (Kunugi et al., 2001; Parsian et al., 2004; Bodner et al., 2005; Vepsäläinen, 2005; Dmitrzak-Weglarz et al., 2008; Borroni et al., 2009; Zdanys et al., 2009; Su et al., 2011; Zhang et al., 2011). Of these BDNF gene polymorphisms, the G196A and C270T SNPs were significantly associated with this diseases in some studies (Kunugi et al., 2001; Ventriglia et al., 2002; Parsian et al., 2004; Olin et al., 2005; Nagata et al., 2011; Dai et al., 2013; Watanabe et al., 2013; Lee and Song, 2014), especially C270T polymorphism was susceptibility to East Asian schizophrenia and AD (Watanabe et al., 2013). Then several researcher replicate this two loci analysis in different diseases, such as PD, schizophrenia, depression patients. ALS, AD, and PD are neurodegenerative diseases that share some common pathways in their onset and progression (Hetz and Mollereau, 2014). Numerous studies have described the overlap of clinical phenotype and gene variants between ALS and schizophrenia (Byrne et al., 2013; Fahey et al., 2014). So we hypothesized that the polymorphisms are susceptible to schizophrenia and neurodegenerative disease may be also susceptible to ALS. So we choose the two widely reported polymorphisms G196A/C270T to analyze its relationship with sALS in a Chinese cohort.

## Subjects and methods

### Participants

This study consisted of 499 sALS patients and 488 healthy control subjects. All participants were of Han Chinese origin. The diagnosis of ALS was made in Peking University Third Hospital (PUTH) between January 2013 and September 2014 using the El Escorial Word Federation criteria for definite or probable ALS (Brooks et al., 2000). This study was approved by the institutional ethics committee of PUTH (IRB00006761), and all patients and controls gave written informed consent.

### SNP analysis

Genomic DNA was collected from peripheral blood leukocytes via standard phenol-chloroform procedures. Genotyping for G196A and C270T BDNF polymorphisms was performed by direct sequencing. The G196A polymorphism was genotyped using the following pair of primers: FW: 5′-ACTCTGGAGAGCGTGAAT-3′ and Rev: 5′-ATACTGTCACACACGCTC-3′. The C270T polymorphism was genotyped using the following pair of primers: FW: 5′-AATGAGACACCCACCGCTGCTG-3′ and Rev: 5′-CTCCTGCACCAAGCCCCATTC-3′. The PCR products were directly sequenced using an ABI3100 automated DNA sequencing system by Tsingke Biotechnology Co., Ltd. (Beijing, China).

### Retrospective observation

We conducted a retrospective review and evaluated both the outcomes and clinical manifestations of these patients. Survival time, which was an endpoint in this study, was defined as the number of months between symptom onset and death from any cause or between symptom onset and tracheostomy for the purpose of permanent mechanical ventilation. “Alcohol abuse” was defined as consuming an alcoholic drink more than twice a week for more than 1 year. “Use of riluzole” was defined as treatment with riluzole (50 mg) twice a day for longer than 2 weeks. The patient characteristics entered in the univariate analyses included age at onset, sex, site (bulbar/spinal) of symptom onset, disease progression ΔFS [progression rate of ALSFRS-R: (48-ALSFRS-R score at first visit)/(time in months between the first symptoms and the first examination)].

### Statistical analysis

Continuous variables were presented as means ± SDs or medians with interquartile ranges, whereas categorical variables were expressed as numbers and percentages. Chi-square test was used to compare group difference for categorical variables. The mean age at onset was compared using Student's *t*-test, while the Mann–Whitney *U*-test was applied to disease progression ΔFS. Univariate and multivariate logistic regressions were used to estimate odds ratios (ORs) and 95% confidence intervals (CIs) for the association between sALS and any of the confounds. In the multivariate logistic regressions, enter method was used with whether ALS or not serves as dependent variable, and gender, age, whether contains risk allele or not as independent variables. The effects of prognostic factors on survival were assessed using the Kaplan–Meier life-table method for all 499 patients according to their risk allele carrier status. The log-rank test was used to assess the equality of the outcome functions. A *P* <0.05 was considered significant after correcting for the number of tests (Bonferroni correction). All analyses were performed using the SPSS V.17.0 software package.

## Results

Of the 499 patients with sALS, the male: female ratio was 1.8:1, and the mean age at onset was 51.29 ± 11.87 years. Other demographic and clinical characteristics were shown in Table 1. Of the 488 healthy control subjects, the male: female ratio was 1.4:1, and the mean age was 52.71 ± 11.81 years in control group. There were no difference between the patients and control subjects in age and gender.

**Table 1:** **Demographic and clinical characteristics of the patients with ALS examined in this study**.

**Demographic and clinical characteristics**	
Phenotype (Total n% from 499 cases)	
Limb-onset ALS	377 (75.56%)
Bular-onset ALS	88 (17.64%)
Flail-arm syndrome (FAS)	30 (6.01%)
Progressive muscular atrophy (PMA)	1 (0.20%)
Primary lateral sclerosis (PLS)	3 (0.60%)
Male (Total n% from 499 cases)	321 (64.32%)
Age at onset (years ± SD)	51.29 ± 11.87
BMI(kg/m2)	23.21 ± 3.28
Smoking	124 (24.84%)
Alcohol abusing	94 (18.84%)
Pesticides	76 (15.23%)
Riluzole using	139 (27.85%)
Treated with non-invasive positive pressure ventilation	44 (8.81%)
Disease progression ΔFS Median [25th, 75th percentiles]	0.72 [0.29–0.89]

*ΔFS: progression rate of ALSFRS-R: (48-ALSFRS-R score at first visit)/(time in months between the first symptoms and the first examination)*.

The two polymorphisms (G196A and C270T) were in Hardy-Weinberg equilibrium in both patients and controls. G196A was not associated with sALS. For C270T, the frequency of the CT genotype and of the T allele was significantly higher in the ALS group than in the control group (Table 2). This difference was significant after Bonferroni correction. The association with T270 was still significant even after adjusting for age and gender *P* = 0.002, OR95%CI = 2.233 [1.353–3.685] (Table 3).

**Table 2:** **Allele and genotype frequencies of the two BDNF SNPs**.

**SNP**	**Samples**	***n***	**Allele (frequency)**		***P* (χ2)**	**Genotype (frequency)**			***P* (χ2)**
			**A**	**G**		**AA**	**AG**	**GG**	
G196A	Cases	499	468 (0.469)	530 (0.531)	0.915 (0.011)	115 (0.230)	238 (0.477)	146 (0.293)	0.267 (2.642)
	Controls	488	460 (0.471)	516 (0.529)		101 (0.207)	258 (0.529)	129 (0.264)	
			**T**	**C**		**TT**	**TC**	**CC**	
C270T	Cases	499	53 (0.048)	945 (0.947)	0.002 (0.018)	0 (0.000)	53 (0.106)	446 (0.894)	0.001 (9.559)
	Controls	488	25 (0.026)	951 (0.974)		1 (0.002)	23 (0.047)	464 (0.951)	

**Table 3:** **Univariate and multivariate logistic regression analysis for variables in sporadic ALS**.

	**Univariate**			**Multivariate**		
**Variable**	**OR**	**95% CI**	***P***	**OR**	**95%CI**	***P***
Gender	1.285	0.994–1.661	0.056	0.791	0.977–1.639	0.075
Age, y	0.991	0.980–1.001	0.083	0.991	0.980–1.001	0.090
T allele in BDNF C270T locus	2.297	1.394–3.786	0.001	2.233	1.353–3.685	0.002

*Variables entered on omnibus tests of Model: gender (1: male, 0: female), age (continuous variable, Years), T allele in BDNF C270T locus (1: with the rest T allele, 0: without the rest T allele)*.

This locus was included in 1000 Genomes project. We can learn that the T allele frequency in Beijing Han Chinese is 2.43% (5/206) from the 1,000 Genomes project database. So we add this data to our control group in order to slightly enlarge our size in the control group. After pooling this data with our control data, the T allele frequency in the new control group is 2.53% (30/1182). The frequency difference between the ALS group and control group was still significant (*P* = 0.0008, OR [95%CI] = 2.154 [1.365–3.398]).

**Figure 1:**
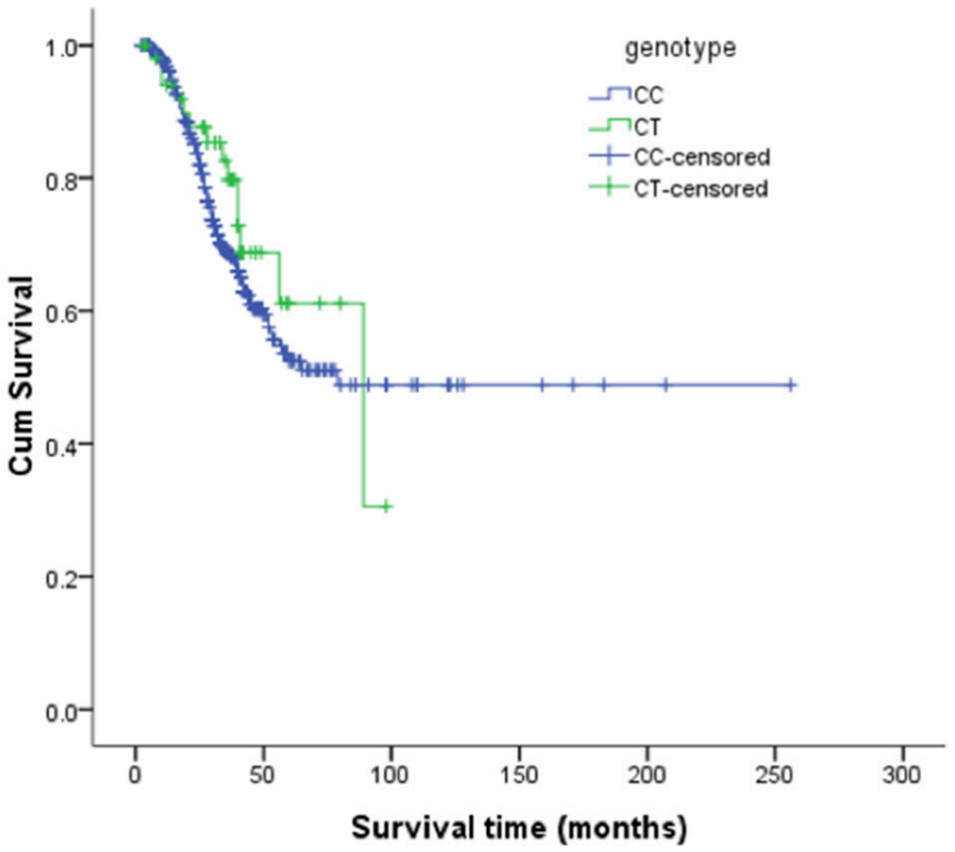
**Kaplan-Meier survival curves**.

The risk allele (T allele; CT genotype) of SNP C270T was detected in 53 of the 499 patients with ALS. We divided the sALS patients into two groups: with and without the T risk allele group. Subgroup analysis was conducted to uncover the association between T risk allele with modifiable exposure and clinical phenotype. There were no difference between the two groups in smoking, alcohol and pesticide exposure, *p*-value respectively was 0.808, 0.480, and 0.977. We could not identify a significant difference in the mean age at onset, sex, site (bulbar/spinal) of onset, disease progression ΔFS, or survival time between the two groups (Table 4). The mean age at onset of ALS was 51.20 (±11.99) years in the patients without the risk allele and 52.02 (±10.89) years in those with the risk allele; this difference was not significant (*t*-test, *P* = 0.630). The sex ratio (male/female) was 284/162 in the patients without the risk allele and 37/16 in those with the risk allele. The site (bulbar/spinal) of symptom onset was 78/368 in the patients without the risk allele and 10/43 in those with the risk allele; again, this difference was not significant (Chi-square test, *P* = 0.803). The disease progression ΔFS also did not significantly differ between the two groups (Mann–Whitney *U*-test, *P* = 0.907). The Kaplan–Meier survival curves of the patients did not depend on C270T T allele carrier status; the curves of the patients with versus without the risk allele did not significantly differ (log-rank test, *P* = 0.316) (Figure 1).

**Table 4:** **Subgroup analysis between T risk allele with modifiable exposure and clinical phenotype**.

**Characteristic**	**Without the risk allele (*N* = 446)**	**With the risk allele (*N* = 53)**	***P*-value**
Sex (male/female)	284/162	37/16	0.449
Mean age at onset (SD) (year)	51.20 ± 11.99	52.02 ± 10.89	0.630
Site of onset (bulbar/spinal)	78/368	10/43	0.803
Disease progression: ΔFS Median (25th, 75th percentiles)	0.54 (0.29–0.88)	0.52 (0.22–0.88)	0.907
Smoking	111/335	14/39	0.808
Alcohol abusing	83/363	12/41	0.480
Pesticides exposure	68/378	8/45	0.977

## Discussion

Here, we provide the first evidence that the BDNF C270T polymorphism might be associated with sporadic ALS in China. Our study suggests that the CT genotype and T allele of C270T in the BDNF gene might increase the risk of sALS in Han Chinese.

Our result is consistent with previous post-mortem and animal studies that have suggested that BDNF plays a role in the pathophysiology of ALS. If the T270 allele itself has a risk-increasing effect, it may result from changes in translation efficacy. The C270T SNP is located in a non-coding region of the 5′-untranslated region (UTR) of the BDNF gene and acts as a functional promoter polymorphism. We searched for potential modifications of transcription factor binding sites in the region of variant using Matinspector prediction software (Cartharius et al., 2005). The analyses indicated that the substitution of allele C with allele T could lend to the loss of transcription factors HINFP and ZIC3 binding sites. As a result, it may alter or control the efficacy of BDNF translation in the somatic, dendritic, or axonal regions of neurons, leading to region-specific quantitative BDNF imbalances in the cortex in patients. Another possibility is that other, currently unknown polymorphisms that are in linkage disequilibrium with the C270T polymorphism confer susceptibility to sALS.

However, we did not find strong evidence of a relationship between the different genotypes and the clinical symptoms of sALS. Altered levels of this neurotrophin have been linked to both cognitive and mood dysfunction (Teixeira et al., 2010). Accordingly, decreased BDNF levels have been reported in patients with depression, and a recent meta-analysis showed that altered peripheral BDNF levels are associated with ongoing depressive disorders (Molendijk et al., 2014). Depressive traits might contribute to major comorbidities and common and overlooked complications that often arise during the course of ALS (Kurt et al., 2007; Thakore and Pioro, 2016; Ye et al., 2016). We did not analyze depression disorders or cognitive function in the patients in this study. The T risk allele may be associated with depression disorders and cognitive function in ALS patients. Clinical trials that evaluate the relationship between genotype and depression disorders and cognitive function in ALS patients will be needed to test this hypothesis.

Our study had some limitations. First, our study just doing SNPs for two loci have some limitations, additional BDNF gene polymorphisms should be included in further study. Second, the sample size of this study was relative small and may be not enough to give a more confirmed conclusion in view of the statistic. However, as ALS is a rare disease, this sample size is relative large for a single center. Further studies with larger sample size especially multiple center studies with different ethnicities should be performed to verify the relationship between BNDF polymorphisms and sALS. Third, the T allele within the patients does not seem to be associated with the clinical phenotype and any modifiable exposure. Our explanation to this phenomena is that the number of the patients with T allele is too small to find any association.

In conclusion, this is the first report about the relationship between BDNF C270T/G196A loci and sporadic ALS. The result of this study indicate that the polymorphism C270T in BDNF gene might be associated with sporadic ALS in China and this relationship should be verified in future studies with larger and more homogeneous samples of different ethnicities.

## Author contributions

DF conceived this study and provided financial support. DF and LX designed the study. LX, DT, and JL took part in the design of the study and in sample collection. DF, LC, and LT conducted data management. LC and LT undertook data checking. LX and DF undertook statistical analysis. DF was responsible for project management. LX and DF were responsible for preparing and revising the manuscript. DF and LX had key roles in the study.

### Conflict of interest statement

The authors declare that the research was conducted in the absence of any commercial or financial relationships that could be construed as a potential conflict of interest.
